# The levels of TGFβ1, VEGF, PDGF-BB, and PF4 in platelet-rich plasma of professional soccer players: a cross-sectional pilot study

**DOI:** 10.1186/s13018-022-03362-4

**Published:** 2022-10-27

**Authors:** Tomoharu Mochizuki, Takashi Ushiki, Satoshi Watanabe, Go Omori, Tomoyuki Kawase

**Affiliations:** 1 Department of Orthopaedic Surgery, Graduate School of Medical and Dental Sciences, Niigata University, Niigata, Japan; 2 Division of Hematology and Oncology, Graduate School of Health Sciences, Niigata University, Niigata, Japan; 3 Department of Transfusion Medicine, Cell Therapy and Regenerative Medicine, Niigata University Medical and Dental Hospital, Niigata, Japan; 4 Department of Hematology, Endocrinology and Metabolism, Faculty of Medicine, Niigata University, Niigata, Japan; 5 Department of Orthopaedic Surgery, Niigata Medical Center, Niigata, Japan; 6 Department of Health and Sports, Faculty of Health Sciences, Niigata University of Health and Welfare, Niigata, Japan; 7 Division of Oral Bioengineering, Graduate School of Medical and Dental Sciences, Niigata University, Niigata, Japan

**Keywords:** Platelet-rich plasma, Athlete, Growth factors, Polyphosphate

## Abstract

**Background:**

Regenerative therapy using platelet-rich plasma (PRP), a rich source of growth factors, has become popular in orthopedic sports medicine. Elite athletes prefer PRP therapy for their injured muscles and tendons primarily to avoid the possible risks of surgical treatment. However, the clinical effectiveness of PRP therapy in elite athletes compared to that in non-athletes remains unknown. Therefore, to investigate the effectiveness of PRP therapy in professional athletes (pro-athletes), we focused on the quality of PRP preparations and compared the levels of bioactive molecules between pro-athletes and non-athletes.

**Methods:**

PRP was prepared from healthy, non-smoking male professional soccer players (pro-athletes) (n = 22) and non-athletes (VEGF: n = 34, others: n = 38). The levels of TGFβ1, PDGF-BB, VEGF, and PF4 were determined using ELISA kits. Polyphosphate was probed with 4’,6-diamidino-2-phenylindole and monitored using a fluorometer. The body composition of the donors was determined using a bathroom weighing scale.

**Results:**

The levels of TGFβ1 and VEGF were significantly lower in pro-athletes than in non-athletes, whereas PF4 levels were significantly higher in pro-athletes. No significant difference was found in PDGF-BB levels between these groups. Biomolecule levels were not correlated with polyphosphate levels.

**Conclusion:**

TGFβ1, VEGF, and PDGF-BB levels in pro-athletes were not higher than those in non-athletes. These findings suggest that growth factor levels in PRP may not be a predominant determinant of the clinical effectiveness of PRP therapy in pro-athletes. Increased PF4 levels in pro-athletes suggest an immunological function of PRP that may positively influence tissue regeneration.

## Introduction

Platelet-rich plasma (PRP) was first reported as a promising tool for regenerative therapy by Marx in the late 1990s [1]. PRP therapy is based on the process of wound healing that involves the release of growth factors from activated, aggregated platelets [2]. It is hypothesized that the efficacy of PRP is due to the amount of growth factors in the individual PRP preparations. Several studies have supported this hypothesis by demonstrating a correlation between the concentrated levels of growth factors, such as platelet-derived growth factor (PDGF) and transforming growth factor β1 (TGFβ1) and platelet counts [2–5]. To date, however, an increasing number of systematic reviews and meta-analyses have reported that the positive effects of PRP application are not only limited to tissue regeneration but also provide symptomatic relief in the treatment of several orthopedic indications, such as rotator cuff tears [6–9], chronic lumber pain, long bone fracture [10, 11], knee osteoarthritis [12], lateral epicondylitis [13], and tendon and ligament [14]. However, a non-negligible number of review articles has expressed skepticism through their meta-analyses of similar orthopedic indications and suggested further randomized clinical trials with qualified PRP preparations in similar clinical indications [15–25]. Such a controversial understanding is due to the quality of individual PRP preparations as well as the condition of individual recipients.

PRP therapy is widely accepted among elite athletes and is the primary choice of medical treatment. The main reason behind this trend is that pro-athletes hope to achieve faster recovery by avoiding surgical risks. Faster recovery could be attributed to early detection and treatment by dedicated medical teams; however, the influence of athlete-specific physical conditions cannot be ruled out by the current understanding. For example, well-trained muscles and superior cardiopulmonary function could contribute to the higher regenerative potential of elite athletes than that of non-athletes.

Athlete-specific physical conditions, i.e., higher physical activity, can be summarized by their higher muscular strength, energy metabolism, motor reflexes, and cardiorespiratory endurance [26, 27]. Interestingly, a recent study indicated that skeletal muscle cells that have had circulating platelets, are known to possess higher energy-metabolic activity than other major nucleated cell types [28]. In this case, because their platelets have higher potential, PRP preparations derived from elite athletes may have higher therapeutic efficacy than those derived from non-athletes. Although reliable cohort studies have not yet been reported, we, like many other sports doctors, have experienced that PRP therapy seems more effective in elite athletes than in non-athletes.

As Marx mentioned in his early publications [29], there is no doubt that the major factors contributing to tissue regeneration are the growth factors stored in platelets. However, we believe that several other factors modulate the therapeutic potential of PRP through potentiation and suppression and are present in individual PRP preparations [30]. The candidate factor that we have recently paid attention to is polyphosphate (polyP) [31–37]. PolyP is an ancient, but still mysterious, biopolymer that is produced in the mitochondria in the extension of the ATP production line [33, 34, 38] and stored in the dense granules of platelets. Upon platelet activation, polyP is released alongside growth factors, and this impacts the immunological functions of platelets in addition to hemostatic function [32]. Thus, polyP could be recognized as a potent bioactive factor as well as a biomarker of platelet energy metabolism, which is the quality of functional platelets. When platelet mitochondria are damaged or dysfunctional, it is speculated that platelets cannot control their response to external stimuli [39, 40]. In the case of knee osteoarthritis, mutations in mitochondrial DNA were proposed as contributors to the risk of progression [41, 42].

To provide evidence for PRP therapy efficacy in elite athletes and to further investigate the possible involvement of polyP in PRP therapy, the levels of bioactive factors (TGFβ1, PDGF-BB, and VEGF) and their correlations with polyP levels in PRP preparations from professional male athletes and male non-athletes of the same age group were compared.

## Materials and methods

### Study design

A cross-sectional study was performed in two independent groups of healthy male Japanese adults (age:19–36 years): the first group (non-athletes: control) comprised ordinary healthy adults (VEGF: n = 34, mean age = 26.0 ± 4.9; others: n = 38, mean age = 26.6 ± 5.1), whereas the second group (pro-athletes: n = 22; mean age = 26.2 ± 4.7) comprised professional soccer players who played in a local team, Albirex Niigata, belonging to the domestic professional soccer league (J. League: https://www.jleague.co/). In the 2022 season, Albirex Niigata won the J2 championship and will automatically be promoted to the J1 league, the top league of the three hierarchized leagues, the next season after six seasons in the J2 league.

The inclusion criteria for the control group were:

- Healthy male young adults
- Non-smokers
- No systemic diseases regardless of medical intervention
- No daily physical training

Exclusion criteria for both groups were:

- Acute or chronic inflammatory conditions reflected in blood cell counts
- Current or former thrombotic or platelet disorders

The indices of body composition and platelet counts of both groups are summarized in Table 1 [34].

**Table 1:** Basic body composition and platelet characteristics of non-athlete and pro-athlete groups

Index	Control (non-athlete)	Pro-athlete	*P*
Age	26.6 ± 5.1	26.2 ± 4.7	0.981*
BMI	23.3 ± 2.5	23.4 ± 1.1	0.375*
BMR	1564.1 ± 121.1	1663.1 ± 96.2	0.00125**
BFP	21.0 ± 3.7	21.8 ± 2.0	0.259**
MPV	9.70 ± 0.65	9.72 ± 0.66	0.890**
TPC	24.9 ± 4.3	23.7 ± 4.7	0.337**

Control (non-athletes) (n = 38) and pro-athletes (n = 22)

^*^Mann–Whitney U test, **Welch’s test (Two-tailed)

BMI, body mass index; BMR, basal metabolic rate; BFP, body fat percentage; MPV, mean platelet volume; TPC, total platelet count

The study design and consent forms for all procedures (approval no. 2021–0126) were approved by the Ethics Committee for Human Participants at Niigata University (Niigata, Japan) and complied with the Helsinki Declaration of 1964 as revised in 2013.

### Blood collection and preparation of platelet-rich plasma

Blood samples were collected in glass vacuum tubes containing acid-citrate-dextrose-formula A (ACD-A; Vacutainer, Becton, Dickinson and Company, Franklin Lakes, NJ, USA) and examined after a 24 h-incubation period, as described previously [34]. From a blood collection tube (approximately 9 mL whole blood including ACD-A), 0.4 mL PRP was prepared using the double-spin method, immediately frozen, and stored at − 80 °C until use.

Pro-athlete blood samples were collected immediately after the regular season when a medical check was performed. Thus, they were free from physical stress such as hard physical training or regular games. Blood collection was done in the afternoon, approximately 2 − 4 h after lunch, and this was conducted once. For non-athletes as well, blood samples were collected in the afternoon.

### Blood cell counting

The total platelet count and mean platelet volume were determined from whole blood and PRP using an automated hematology analyzer (pocHiV-diff, Sysmex Corporation, Kobe, Japan), as described previously [34].

### Determination of body Composition

Prior to blood collection, the body composition of donors was determined using a bathroom weighing scale (HCS-FS03; ECLEAR, ELECOM, Osaka, Japan), which was installed with a unique MRI-based program [43] that enables a more accurate determination of an individual’s body mass index, body fat percentage, and basal metabolic rate based on measured body weight and bioelectrical impedance [34].

For body composition measurement, because of the direct measurement-based evaluation, densitometry has gained widespread use and was considered as “gold standard” until recently [44, 45]. Compared with these modalities, the measurement reliability of bioelectrical impedance analysis (BIA) is influenced by various factors, such as electrodes, operators, subjects, and the environment [46]. However, owing to its advantages of speed, non-invasiveness, inexpensiveness, and portability, BIA currently seems to be the most feasible body composition measurement technique for bedside use [47] and was suitable for our measurement conditions.

### Quantification of platelet polyP levels

As previously described [32, 35], platelets fixed with ThromboFix (Thermo Fisher) were probed in Milli-Q water containing 4 μg/mL DAPI (Dojin, Kumamoto, Japan), and the fluorescence intensity was measured using a fluorometer (FC-1; Tokai Optical Co., Ltd.) with excitation and emission wavelengths of 425 and 525 nm, respectively.

### Determination of growth factor and cytokine levels by ELISA

As previously described [48], the concentrations of TGF-β1, platelet-derived growth factor-BB (PDGF-BB), and platelet factor 4 (PF4) in frozen PRP were determined using human TGF-β1, PDGF-BB, and PF4 Quantikine ELISA kits (R&D Systems, Inc., Minneapolis, MN, USA). Concentrations of vascular endothelial growth factor (VEGF) were determined using the LBIS Human VEGF ELISA Kit (FUJIFILM Wako Chemicals, Osaka, Japan).

### Statistical analysis

**Fig. 1:**
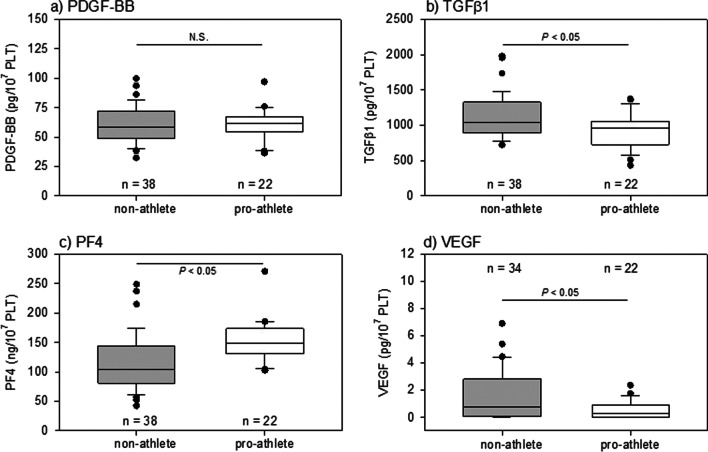
Levels of PDGF-BB, TGFβ1, PF4, and VEGF in PRP preparations from young non-athletes and pro-athletes

**Fig. 2:**
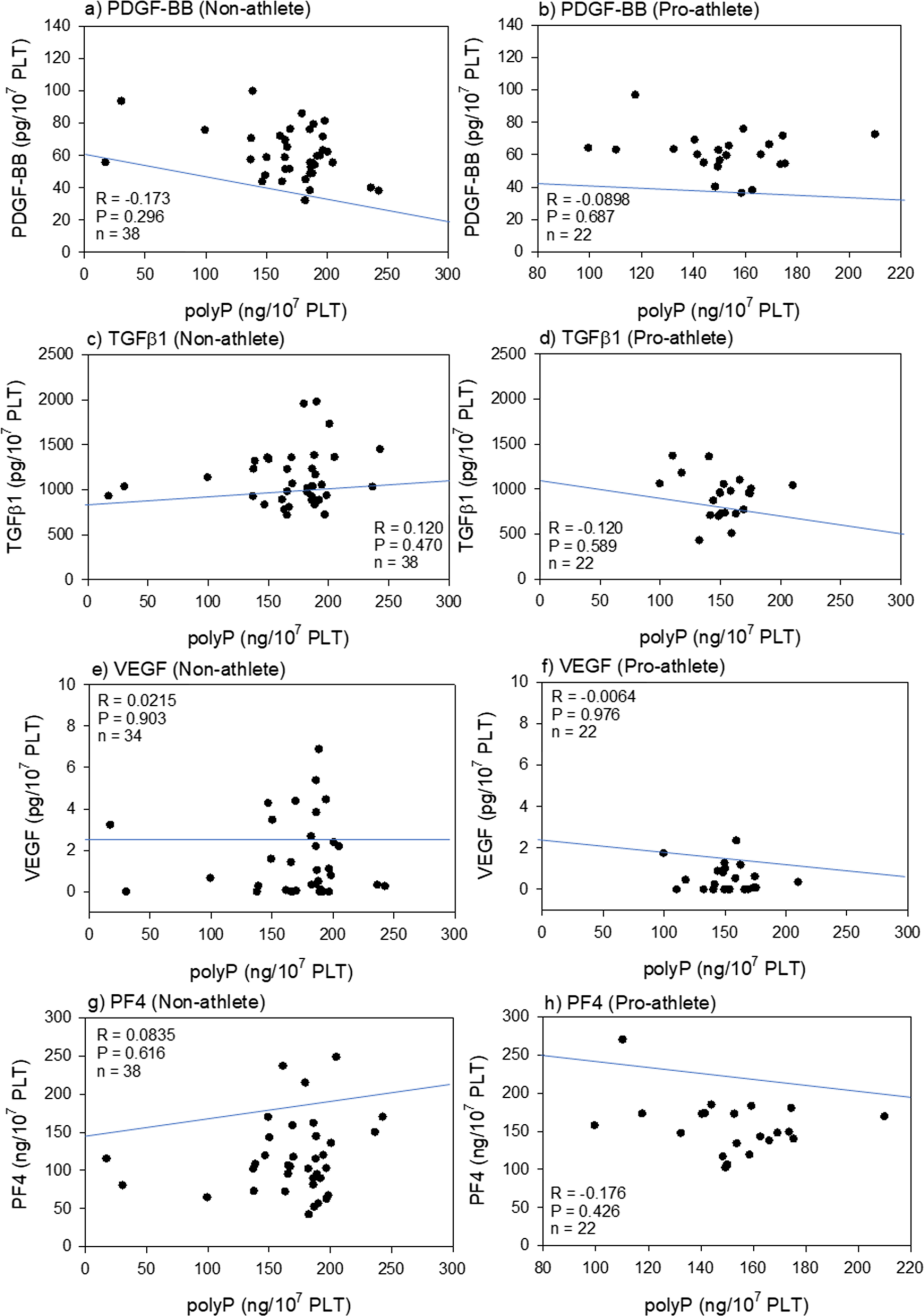
Correlations between polyP levels and PDGF-BB (**a**, **b**), TGFβ1 (**c**, **d**), VEGF (**e**, **f**) or PF4 levels (**g**, **h**) in the non-athlete (**a**, **c**, **e**, **g**) and the pro-athlete (**b**, **d**, **f**, **h**) groups. polyP, polyphosphate

To compare each biomolecule level between the two groups, the data are expressed as box plots in Fig. 1. Body indices are expressed as the mean ± SD in Table 1. Mann–Whitney U test or Welch’s t-test was performed to confirm statistical differences (SigmaPlot version 14.5; Systat Software, Inc., Systat Software, Inc., San Jose, CA, USA). Differences were considered statistically significant at *P* < 0.05. Spearman’s correlation analysis was performed to compare each correlation between the two indices, and correlation coefficients were calculated using SigmaPlot software (Fig. 2). Differences were considered statistically significant at *P* < 0.05.

## Results

We compared the levels of representative bioactive factors per unit platelet count in PRP preparations derived from pro-athletes and non-athletes. As shown in Fig. 1, the levels of TGFβ1 and VEGF were higher in non-athletes than those in pro-athletes, whereas those of PF4 were higher in pro-athletes. No significant differences were found in PDGF-BB levels between these groups.

The correlation between bioactive factor levels and polyP levels were analyzed in pro-athletes and non-athletes. In our working hypothesis, because bioactive factors stored in intra-platelet granules are released along with polyP upon activation, the levels of bioactive factors are assumed to correlated with those of polyP. However, as shown in Fig. 2, no moderate or strong correlations were found between any of the bioactive factors.

## Discussion

To our knowledge, this is the first report to compare the bioactive factor levels in PRP between elite athletes and non-athletes. In anuclear platelets, bioactive factors are released from intracellular storage sites, such as α-granules [49], without new synthesis from genomic DNA. Blood samples were collected during medical checks for pro-athletes and at intervals between business or classes for non-athletes; thus, the influence of heavy acute physical or mental strain could be excluded. Therefore, it is conceivable that the differences in bioactive peptide factor levels (TGFβ1, VEGF, PDGF-BB, and PF4) are due to the original storage levels or tolerance variability among individuals.

### Growth factors and PF4 storage in platelet α-granules

Storage levels are primarily determined by protein synthesis from genomic DNA in megakaryocytes. As this is a fundamental aspect, we have not discussed this in detail in this study. However, platelet behavior may be influenced by general conditions such as physical trauma, strain, or injury, Platelet sensitivity to external stimuli may be higher in pro-athletes than in non-athletes as the former is more prone to develop venous thromboembolism [50, 51]. During physical stress the sympathetic nervous system enters a state of dormancy, and circulating platelets are potentially exposed to adrenergic stimuli, leading to aggregation and activation with an increased release of stored growth factors [52–54]. Consequently, stored growth factor levels decrease.

Decreased levels of TGFβ1 and VEGF confirm this scenario; however, no statistical difference in PDGF-BB was identified. This unexpected result may be due to the dimeric configuration of PDGF. PDGF-BB is an isoform of the five dimeric isoforms derived from the four gene products in the PDGF family [55]. Therefore, for a more accurate comparison, the total levels of all these isoforms need to be determined, wherein differences, similar to those in TGFβ1 and VEGF, could be detected.

In contrast, PF4 levels were significantly higher in pro-athletes than in non-athletes, despite being stored in α-granules like TGFβ1 and VEGF [56, 57]. The differences between PF4 and growth factors are the much higher levels of storage and the recycling system at the megakaryocyte stage [56, 57]. Immune function is suppressed in pro-athletes [58]; however, it is possible that this hypofunction is compensated for by the upregulated network between platelets and other immune cells through the intermediation of increased levels of PF4. As further information is not currently available in literature, it is interesting to establish this phenomenon and clarify the mechanism for a better understanding of the possible immunological functions of PRP.

### Polyphosphate stored in dense granules

Polyphosphate was vigorously studied and discussed in the 1980s and 90s [36, 37], however, the role of eukaryotic polyP as a bioactive factor remains unclear. All of the suggested functions, except the hemostatic action of polyP, have not yet been clearly demonstrated [59]. Nevertheless, a recent study demonstrated that PF4 forms complexes with polyP and thereby activates platelets through an autocrine loop [60], suggesting a synchronized behavior of these factors. However, we found in the previous [34] and current studies demonstrate that PF4 and polyP behave differently. Considering another possible function of platelet polyP as an alternative energy stock [34], the “seemingly-conflicting” discrepancy between PF4 and polyP levels may be acceptable but needs to be further studied to precisely evaluate the quality of PRP.

To our knowledge, the involvement of polyP in orthopedic and sports medicine is poorly understood. However, in addition to studies implying the involvement of mutated mitochondrial DNA in exacerbated knee osteoarthritis [61], some review articles indicated a relationship between age-related mitochondrial dysfunction and sarcopenia [62, 63]. Thus, considering the correlation between platelet and muscle energetics, even though polyP is not directly involved in muscle function, it might be helpful to evaluate the status of mitochondria through their products, i.e., polyP and ATP, and DNA for the diagnosis of recipient body conditions, identification of responder patients for PRP therapy, and eventual establishment of personalized treatment algorithms in the future [61].

### Limitations

This is a pilot study that requires further investigation. First, the sample size, especially of pro-athletes, was too small to perform statistical analysis. Second, blood collection from the same donors was not repeated to determine the reproducibility or diurnal variation of the data. Thus, the data may not necessarily reflect the actual status or reality. However, the protocol of blood collection from this study correlates with autologous PRP preparations used in clinical settings as these are derived from one-time collected samples. Third, for several reasons, the study adopted the BIA technique to measure body composition. However, the BIA technique is inferior to densitometry and other X-ray-based techniques in terms of accuracy and reliability. Thus, to further explore the relationship between PRP quality or recipient conditions and body composition, gold-standard techniques should be adopted to acquire more reliable data.

### Prospective view and further studies

Regenerative therapy using PRP has become widespread over the last two decades. However, it is difficult to appreciate that the clinicians’ understanding has reached a standard level, as it notably varies among individuals [64]. Using a series of original and review articles [65–70], this study proposed important factors to focus on for better PRP therapy. However, to the best of our knowledge, no breakthrough advances have been achieved in this decade.

The goal of this study is to make PRP therapy more predictable by improving the diagnostic procedure to discern the responder from the non-responder to save athletes’ careers. Thus, we plan to overcome the previously discussed limitations using a step-by-step approach and combine basic research with clinical research to analyze the possible correlation between PRP quality and clinical outcome. Simultaneously, reliable cohort studies that address the longstanding question by comparing the responsiveness of pro-athletes to PRP therapy with that of non-athletes are essential.

### Clinical relevance

**Fig. 3:**
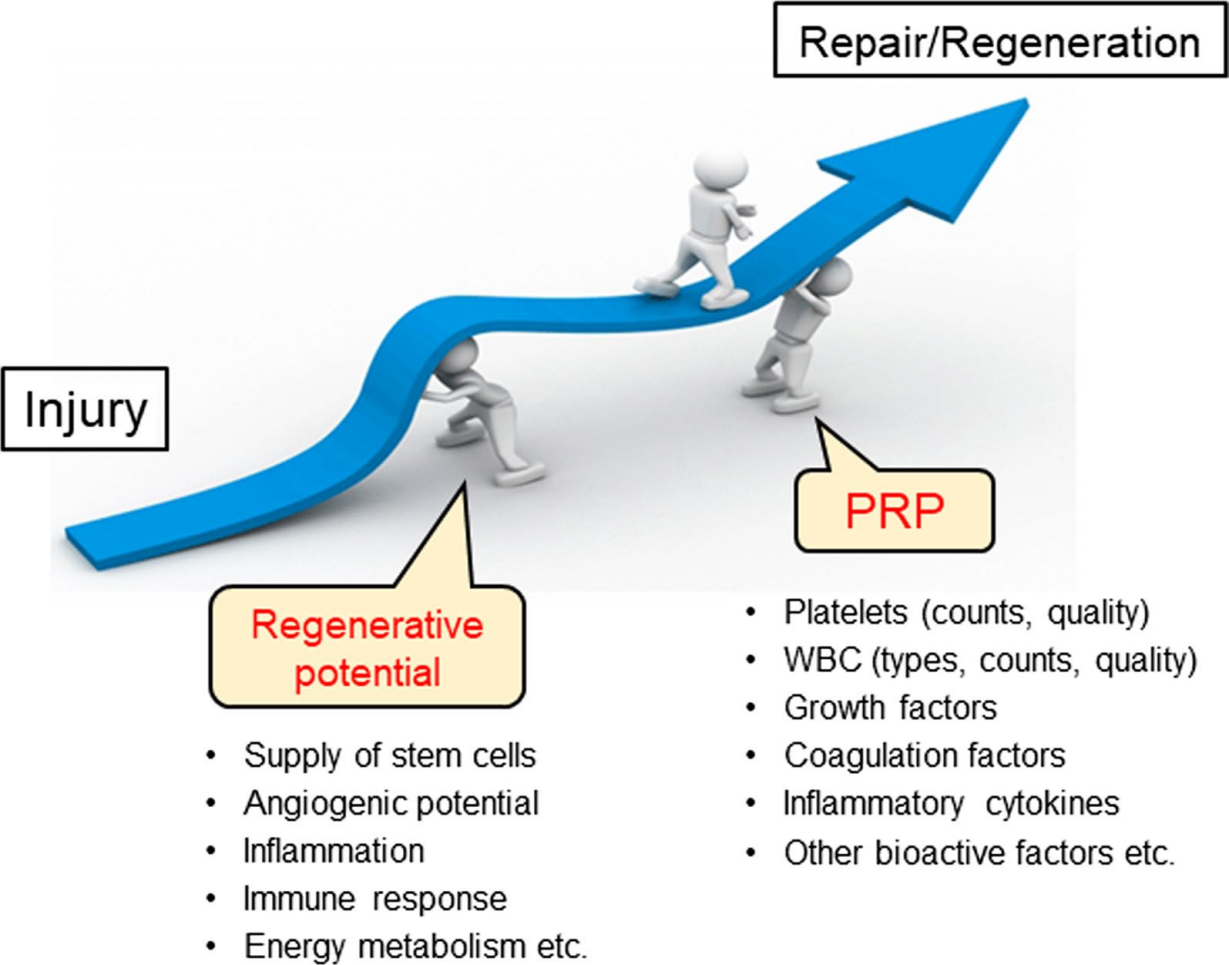
Proposed relationship, the “adjuvant principle”, between recipient’s regenerative potential and PRP therapy during tissue repair and regeneration

According to clinical experience, many sports doctors have a similar impression that PRP therapy enables elite athletes to return from medical leave due to injury faster than non-athletes or non-professional (amateur athletes). If this is true, unlike the widely accepted understanding of PRP therapy, growth factor levels are not the predominant determinant of clinical outcomes. As previously claimed [65], the outcome of PRP therapy can be determined by the balance between recipient conditions and PRP quality based on the adjuvant principle (Fig. 3) [65]. It is assumed that PRP therapy works successfully only when the recipient’s condition, especially the regeneration activity, is maintained at moderate to high levels. If the recipients’ regenerative activity is too low, for example, in elderly individuals, the expected outcome of PRP therapy could not be induced. However, it should be noted that if the recipients’ regenerative activity is sufficiently high, for example in younger schoolchildren, the expected outcome of PRP therapy may be hidden by their spontaneous regenerative response.

A similar concept has been proposed more specifically by Maffulli and his coworkers [61, 71, 72], and the clinical effectiveness of PRP is a result of its interaction with recipient tissue. The status of the recipient tissue, primarily demographic factors, immune status, metabolic diseases, and concomitant medications, could influence the effectiveness of PRP. Nevertheless, the possible relationships between these factors and PRP effectiveness have been poorly explored and remains to be established. It is important for clinicians to acquire the ability to predict which patients will respond positively to PRP. Thus, we again propose that further attention should be paid to recipients’ conditions to obtain an in-depth understanding of PRP therapy [65].

## Conclusion

This pilot study suggests that growth factor levels in individual PRP preparations may not be a predominant determinant of the clinical efficacy of PRP therapy, especially in pro-athletes. Increased PF4 levels in pro-athletes suggest an immunological function of PRP that may positively influence tissue regeneration.

## Data Availability

The data are available from the corresponding author on reasonable request.
